# FEOpti-ACVP: identification of novel anti-coronavirus peptide sequences based on feature engineering and optimization

**DOI:** 10.1093/bib/bbae037

**Published:** 2024-02-14

**Authors:** Jici Jiang, Hongdi Pei, Jiayu Li, Mingxin Li, Quan Zou, Zhibin Lv

**Affiliations:** College of Biomedical Engineering, Sichuan University, Chengdu 610065, China; College of Biomedical Engineering, Sichuan University, Chengdu 610065, China; College of Life Science, Sichuan University, Chengdu 610065, China; College of Biomedical Engineering, Sichuan University, Chengdu 610065, China; Institute of Fundamental and Frontier Sciences, University of Electronic Science and Technology of China, Chengdu 610054, China; Yangtze Delta Region Institute (Quzhou), University of Electronic Science and Technology of China, Quzhou 324000, China; College of Biomedical Engineering, Sichuan University, Chengdu 610065, China

**Keywords:** anti-coronavirus peptides, feature engineering, synthetic minority over-sampling technique, machine learning, UniRep, light gradient boosting

## Abstract

Anti-coronavirus peptides (ACVPs) represent a relatively novel approach of inhibiting the adsorption and fusion of the virus with human cells. Several peptide-based inhibitors showed promise as potential therapeutic drug candidates. However, identifying such peptides in laboratory experiments is both costly and time consuming. Therefore, there is growing interest in using computational methods to predict ACVPs. Here, we describe a model for the prediction of ACVPs that is based on the combination of feature engineering (FE) optimization and deep representation learning. FEOpti-ACVP was pre-trained using two feature extraction frameworks. At the next step, several machine learning approaches were tested in to construct the final algorithm. The final version of FEOpti-ACVP outperformed existing methods used for ACVPs prediction and it has the potential to become a valuable tool in ACVP drug design. A user-friendly webserver of FEOpti-ACVP can be accessed at http://servers.aibiochem.net/soft/FEOpti-ACVP/.

## INTRODUCTION

Coronaviruses contain a positive-strand RNA genome that, ranging from 27 to 32 kb, is the largest of all known RNA viruses [1]. Once the virus enters host cell, a series of non-structural proteins form a large supramolecular protein structure referred to as the replication and transcription complex (RTC). Containing polymerase, primase, helicase, methyltransferase and nuclease enzymes, as well as various cofactor proteins, are essential for the core process of viral transcription and replication. Not surprisingly, it contains several potential targets for antiviral drug design [2]. Severe acute respiratory syndrome-associated coronavirus, SARS-CoV-2, was discovered in late 2019 [3]. It belongs to the beta coronavirus genus within the Coronaviridae family. The pandemic caused by this virus killed millions of people globally and led to lasting health problems in a proportion of patients who recovered. Beta coronaviruses were also responsible for the severe acute respiratory syndrome (SARS) epidemic in 2003 and the outbreak of middle east respiratory syndrome (MERS) in 2012. Given this history, it is essential to develop novel strategies to combat infections cause by this class of viruses.

The difficulty of developing small molecule drugs, combined with the growing maturity of genetic engineering and solid-phase peptide synthesis technology, resulted in the emergence of therapeutic peptide drugs [4–9]. This interest is also fueled by the wide adaptability, high safety and remarkable efficacy of this approach. In particular, peptide-based pan-coronavirus fusion inhibitors can effectively inhibit infections by SARS-CoV-2 and its variants, as well as other human coronaviruses [10–16]. Although laboratory experiments can identify novel anti-coronavirus peptides (ACVPs), this process is expensive and time consuming. Therefore, there is an increasing interest in the use of computational approaches to predict peptides with potential ACVPs activity.

Several methods have been developed to predict the sequence of potential ACVPs. In 2021, Pang et al. [17] described a two-stage classification PreAntiCoV approach, taking into account the amino acid composition (AAC), dipeptide composition (DiC), the composition of k-spaced amino acid group pairs (CKSAAGP), pseudo amino acid composition (PAAC) and physicochemical features (PHYC) to form peptide descriptors using a balanced random forest (RF) dataset. In the same year, Timmons et al. [18] proposed ENNAVIA to predict antiviral and ACVPs. This approach used amino acid and other physicochemical descriptors for feature extraction and combined these with machine learning (ML) utilizing deep neural networks, which achieved better predictive functionality than PreAntiCov [19]. In 2022, Kurata et al. [20] introduced iACVP, an approach that used a word embedding model, word2vec (W2V), to extract peptide sequence features. This was combined with several ML methods, including transformer, convolutional neural network (CNN), bidirectional long short-term memory (BiLSTM), RF and support vector machine (SVM) to develop classifiers [21–24]. In 2023, Chen et al. [25] proposed PACVP, a peptide prediction strategy that employed nine peptide sequence characteristics to extract relevant features identifying potential ACVPs. These included the previously tried AAC, CKSAAGP, PAAC and DiC. In addition, they also incorporated adaptive skip dipeptide composition (ASDC), the composition transition and distribution (CTD) tool, quasi-sequence order descriptor (QSO), amino acid entropy (AAE), Boruta, multi-resolution multi-directional (MRMD) analysis and a tree-based algorithm (SFM-XGBoost). This group also tested five ML methods and superposition learning during the development of their peptide predictor. This expansion of the analyzed characteristics brought some benefits in improving the accuracy in ACVPs prediction. While all of the above approaches achieved reasonable performance in tests, they still failed to meet the needs of drug development. The fact that only a relatively limited number of experimentally validated ACVP sequence data was available for model training and development led to an imbalanced benchmark dataset. To obtain more information about relevant sequence features, the use of additional computational frameworks, including deep learning and natural language processing, was suggested [26–29].

Deep learning learns the underlying patterns in large datasets by understanding the laws and layers of sample data to replicate human analytical and learning abilities through artificial intelligence. A range of deep learning-based computational approaches has been used widely in bioinformatics, allowing machines to identify features of protein or peptide sequences and transform these to represent relevant information about the object of interest [30–37]. Effective sequence representation approaches include soft-alignment mechanism (SSA) [38], unified representation (UniRep) [38–40], BiLSTM [41] and bidirectional encoder representation from transformers (BERT) [42].

In this study, we describe the development of a novel approach to predict ACVPs, FEOpti-ACVP, that is based on feature engineering (FE) optimization. Since the existing benchmark dataset of identified ACVPs is imbalanced, the synthetic minority over-sampling technique (SMOTE) was utilized to improve peptide representation. The resulting feature vectors and their fused representations, optimized by the embedding feature selection method, were entered into seven ML models to construct the new ACVPs predictor. In addition, we took advantage of the unified manifold approximation and projection (UMAP) algorithm to visualize optimized feature vectors and to compare model performance. The results showed that FEOpti-ACVP demonstrated superior performance for the prediction of ACVPs in both 5-fold cross-validation (ACC = 0.992, MCC = 0.984, Sn = 0.998, Sp = 0.986, auROC = 1.000, auPRC = 1.000 and F1 = 0.992) and in independent tests (ACC = 0.967, MCC = 0.742, Sn = 0.656, Sp = 0.992, auROC = 0.927, auPRC = 0.729 and F1 = 0.789). In comparison with existing prediction models, FEOpti-ACVP showed better overall performance and has the potential to be applied widely in the discovery of novel ACVP drugs.

## MATERIALS AND METHODS

### Benchmark dataset

To facilitate further comparisons, the model in this paper was developed using the latest dataset from PACVP [25, 43, 44]. This ACVP-2141 benchmark dataset contains 157 ACVP and 1984 peptides with no demonstrable anti-coronaviral activity (named non-ACVP). The dataset was ‘de-homologized’ by removing sequences with >90% identity. For the purposes of training and testing our model, this dataset was arbitrarily partitioned at a 4: 1 ratio to construct a training dataset, ACVP-TR, and the test dataset, ACVP-IND.

### Overview of FEOpti-ACVP

**Figure 1:**
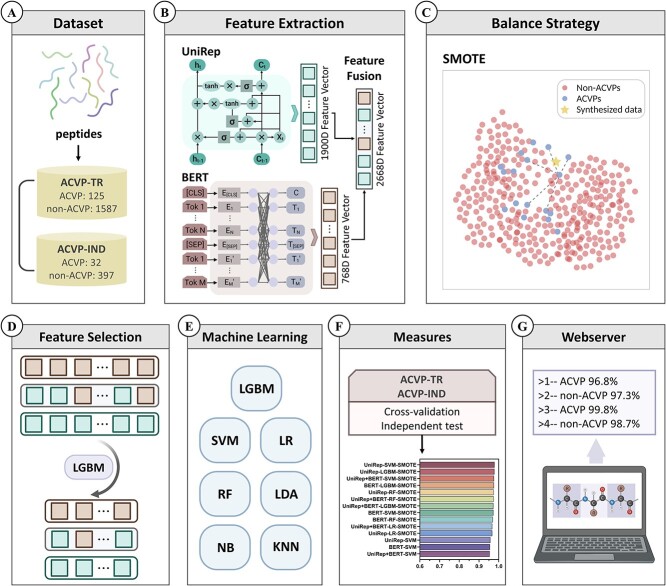
Schematic diagram of model development. (**A**) Construction and partitioning of benchmark dataset. (**B**) Feature vector extraction using the pre-trained UniRep and BERT sequence embedding models. This extraction was conducted twice, creating either the 1900-dimensional(1900D) UniRep feature vectors or the 768-dimensional BERT feature vectors. These were also fused to obtain the 2668-dimensional UniRep+BERT feature vectors. (**C**) The SMOTE model was used to balance the heavily skewed representation of ACVP and non-ACVP sequences in the original dataset. (**D**) Using the LGBM algorithm, the extracted feature vectors were ranked according to their importance, to eliminate redundant features. (**E**) Testing of the feature selected dataset as input into the seven tested ML methods: LGBM, SVM, LR, RF, LDA, KNN and NB. (**F**) Evaluate the best performing model based on the results of metrics achieved using the independent test sequence dataset. (**G**) Developing a web server FEOpti-ACVP based on the final optimized model.

FEOpti-ACVP’s development strategy is in Figure 1. The process involved the construction of the benchmark dataset, feature extraction, synthesis of features to balance the dataset, feature selection, ML model training, 5-fold cross-validation, independent testing, and finally, constructing the FEOpti-ACVP webserver.

### Synthetic minority over-sampling technique

As mentioned in Section 2.1, the negative dataset is ~13 times larger than the positive dataset. One approach to address class imbalance in datasets is to use the SMOTE [45]. Here’s how it works: for each sample $x$ in the minority class, calculate its Euclidean distance with all other samples in the class. Then, select the value of $k$ ($k$ is a parameter) to determine the number of nearest neighbors of the sample. From these neighbors, choose one (for example, $y$) randomly to generate a new sample $z$. The formula for generating $z$ is


(1) \begin{equation*} z=x+\lambda \times \left(y-x\right) \end{equation*}

Here, $\lambda$ is a random number between 0 and 1. This process is repeated until the desired balance between the minority and majority classes is achieved.

### Feature extraction

In the pre-training stage of model development, feature extraction is an effective way of strengthening data representation, since the obtained new features map onto the original features. We used both the UniRep and BERT for the purposes of feature extraction to pre-train our model on the ACVP-2141 dataset. The obtained feature vectors, processed by the two distinct algorithms, were then used as the input for ML model training and comparison.

#### The UniRep feature extractor

Alley et al. [46] developed a deep learning-based sequence representation method, UniRep, a one-way mLSTM with 1900 hidden features. The authors used a recurrent neural network (RNN) to compile the statistical representation of proteins, resulting in a unified representation that integrates arbitrary protein sequences as fixed-length vectors that approximate underlying protein features. Although the fixed hidden state of RNNs can result in a bottleneck, the UniRep approach demonstrated that RNNs could adequately use raw sequence data for the task of training an algorithm to predict the next amino acid in a sequence. Thus, using UniRep could alleviate data scarcity that previously constrained protein informatics.

#### The BERT feature extractor

Devlin et al. [47, 48] introduced a pre-trained language representation model, BERT. It uses a masked language model (MLM) to pre-train bidirectional transformers, where each token attends to ‘all tokens’. After stacking multiple layers of transformer structures, the main framework of the BERT is created by stacking multiple layers of transformer structures. The model ultimately provides a deep bidirectional linguistic representation capable of incorporating contextual information from any direction.

Firstly, the ACVP sequence is converted into a token representation of k-mer, and a token representation position is added to obtain the input token. Then, the context semantics are captured by the multi-head self-attention model, and a specific adjustment is obtained through linear transformation, thus ending the forward propagation of one layer. Through multiple such layers in the model (as shown in Figure 1), the final results are used for the BERT pre-training task.

### Feature selection

Feature selection, an indispensable step in building predictive models, is the process where significant features are identified and retained, while redundant/uninformative features are discarded. This process alleviates the issue of excessive dimensionality, and reduces the risk of overfitting, thus improving model performance. Filter, wrapper and embedded are three methods used in feature selection. During the creation of our model, we employed embedded to obtain feature importance and perform feature selection while the model was being trained. We introduced a light gradient boosting machine (LGBM) step to construct an iterative boosting tree model [49], covering the original features, creating weight coefficients for each feature. Features were selected according to this weight coefficient, ranking them from largest to smallest.

### Machine learning

Supervised learning is a training technique in ML where the algorithm uses a dataset of known categories to adjust classifier parameters to build a learning model that achieves the target performance. The categorization of new instances is predicted based on this trained model. Regardless of learning style or function, all ML algorithms involve the following elements: representation, evaluation and optimization. In this paper, seven supervised learning algorithms were tested, comparing their performance, to select the best option to build the final model. The tested algorithms included LGBM [41], RF [33], SVM [29], k-nearest neighbors (KNN) [42], linear regression (LR), latent dirichlet allocation (LDA) [38] and naive bayes (NB) [50], which have been proven effective in other peptide/protein predictions.

### Performance measures

During the development of model, we relied on seven metrics commonly used to evaluate performance: accuracy (ACC), Matthews correlation coefficient (MCC), sensitivity (Sn), specificity (Sp), F1-score (F1), area under the receiver operating characteristic curve (auROC) and area under the precision-recall curve (auPRC) [51, 52]. F1 is a reconciled average of precision and recall. In general, auPRC and F1 are also considered objective measures of the performance of models developed using imbalanced datasets [53]. The formulas used to derive the above metrics are as follows:


(2) \begin{equation*} ACC=\frac{TP+ TN}{TP+ TN+ FP+ FN} \end{equation*}

(3) \begin{equation*} MCC=\frac{TP\times TN- FP\times FN}{\sqrt{\left( TP+ FP\right)\left( TP+ FN\right)\left( TN+ FP\right)\left( TN+ FN\right)}} \end{equation*}

(4) \begin{equation*} Sn= Recall=\frac{TP}{TP+ FN} \end{equation*}

(5) \begin{equation*} Sp=\frac{TN}{TN+ FP} \end{equation*}

(6) \begin{equation*} Precision=\frac{TP}{TP+ FP} \end{equation*}

(7) \begin{equation*} F1=\frac{2\times Precision\times Recall}{Precision+ Recall} \end{equation*}

where TP (true-positive) is the number of correctly identify positive ACVPs, TN (true-negative) is the number of correctly identified non-ACVPs, FP (false-positive) is the number of ineffective peptides incorrectly classified as ACVPs and FN (false-negative) is the number of ACVPs incorrectly identified as non-ACVPs.

We employed 5-fold cross-validation and independent tests were to evaluate these models. For the purposes of testing, the ACVP-TR dataset was subdivided into five subsets, four of these were used for training, while the last subset is used in the final testing of the model. The average of the five tests is used as the final result of the 5-fold cross-validation [54, 55]. Independent testing used the ACVP-IND data subset that did not overlap with the training dataset. Thus, samples used for independent testing were new to the model.

## RESULTS AND DISCUSSION

### SMOTE expanded dataset promotes better model performance

Since the number of peptides with confirmed ACVP activity is currently limited, any training dataset is skewed by the overrepresentation of peptides with no activity, causing an imbalance (Figure 4A–C). To improve the effectiveness of the pre-trained model, we introduced SMOTE to improve the balance of the dataset (Figure 4D–F). The results of the 5-fold cross-validation and independent testing achieved using the SMOTE generated dataset are shown in Supplementary Table 1, while the visualization of feature representations by UMAP is shown in Figure 4.

**Figure 2:**
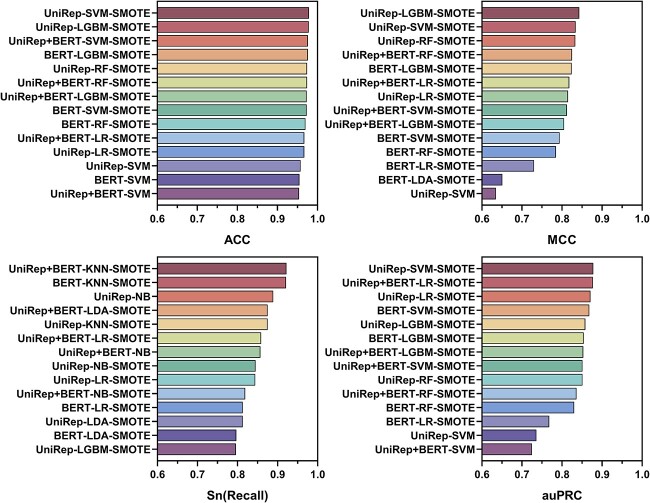
Top 14 of 5-fold cross-validation and independent test results were averaged from runs conducted with and without SMOTE-based feature augmentation/balancing. Performance metrics were ranked from best to worst after completing a total of 42 runs.

As shown in Figure 2, we ranked the average of the 5-fold cross-validation and independent test results according to different metrics, including ACC, MCC, Sn and auPRC. Sn (recall) and auPRC are recognized as efficiency metrics in evaluating imbalanced data. In the top 14 rankings of ACC, MCC, Sn and auPRC, the proportion of SMOTE models accounted for 78.57, 92.86, 85.71 and 85.71%, respectively. Therefore, introducing SMOTE as a balancing strategy was a powerful way to improve model performance. The following sections discuss data based on this balancing strategy.

### The effect of feature fusion on prediction performance

In an attempt to obtain more representative feature information, we fused the 1900D UniRep feature vectors with the 768D BERT feature vectors, obtaining a 2668D combined UniRep+BERT feature vector set and tested the predictive performance of all three vector sets. The results of the 5-fold cross-validation and independent testing are shown in Table 1.

**Table 1:** Results of the 5-fold cross-validation and independent testing of the three feature models developed using UniRep and BERT feature extraction. The used ML are indicated on the right

Model	Feature	5-fold cross-validation								Independent test						
		ACC	MCC	Sn	Sp	auROC	auPRC	F1		ACC	MCC	Sn	Sp	auROC	auPRC	F1
LGBM^a^	UniRep^b^	0.994	0.987	**0.999** ^c^	0.989	**1.000** ^d^	**1.000**	0.994		**0.963**	**0.698**	**0.594**	**0.992**	0.922	**0.716**	**0.742**
	BERT^b^	**0.995**	**0.989**	**0.999**	**0.991**	**1.000**	**1.000**	**0.995**		0.958	0.658	0.563	0.990	**0.926**	0.708	0.715
	UniRep+BERT^b^	0.992	0.985	0.998	0.987	**1.000**	**1.000**	0.992		0.953	0.626	0.563	0.985	0.925	0.706	0.713
SVM^a^	UniRep^b^	**0.998**	**0.996**	0.999	**0.996**	**1.000**	**1.000**	**0.998**		**0.960**	**0.672**	**0.531**	**0.995**	**0.934**	**0.756**	**0.692**
	BERT^b^	0.992	0.984	**1.000**	0.984	**1.000**	**1.000**	0.992		0.953	0.604	0.469	0.992	0.929	0.737	0.635
	UniRep+BERT^b^	0.997	0.994	0.999	0.995	**1.000**	**1.000**	0.997		0.956	0.629	0.500	0.992	0.926	0.703	0.663
RF^a^	UniRep^b^	0.986	0.971	0.985	0.986	**0.999**	**0.999**	0.986		**0.963**	**0.695**	**0.563**	**0.995**	0.918	**0.704**	**0.718**
	BERT^b^	**0.988**	**0.977**	**0.989**	0.988	**0.999**	**0.999**	**0.988**		0.951	0.594	0.500	0.987	0.923	0.661	0.661
	UniRep+BERT^b^	0.987	0.975	0.985	**0.990**	**0.999**	**0.999**	0.987		0.960	0.676	**0.563**	0.992	**0.926**	0.673	0.717
LR^a^	UniRep^b^	0.978	0.956	**1.000**	0.955	0.995	0.993	0.978		**0.956**	**0.675**	0.688	**0.977**	0.940	0.750	0.804
	BERT^b^	0.974	0.950	**1.000**	0.948	0.990	0.983	0.975		0.923	0.511	0.625	0.947	0.885	0.553	0.745
	UniRep+BERT^b^	**0.981**	**0.964**	**1.000**	**0.963**	**0.999**	**0.998**	**0.982**		0.953	0.672	**0.719**	0.972	**0.941**	**0.756**	**0.823**
KNN^a^	UniRep^b^	0.894	0.806	**1.000**	0.787	0.946	0.902	0.904		0.786	0.326	0.750	0.788	0.807	0.279	0.765
	BERT^b^	**0.902**	**0.820**	0.997	**0.807**	**0.952**	**0.913**	**0.911**		**0.809**	**0.398**	**0.844**	**0.806**	0.885	**0.385**	**0.828**
	UniRep+BERT^b^	0.891	0.801	**1.000**	0.782	0.937	0.889	0.902		0.776	0.362	**0.844**	0.771	**0.873**	0.297	0.814
LDA^a^	UniRep^b^	0.888	0.797	**1.000**	0.777	0.901	0.835	0.900		0.779	0.256	0.625	0.791	0.742	0.166	0.682
	BERT^b^	**0.945**	**0.896**	**1.000**	**0.890**	**0.987**	**0.971**	**0.948**		**0.890**	**0.406**	0.594	**0.914**	**0.858**	**0.452**	0.707
	UniRep+BERT^b^	0.904	0.823	**1.000**	0.807	0.938	0.890	0.912		0.804	0.347	**0.750**	0.809	0.807	0.219	**0.773**
NB^a^	UniRep^b^	0.780	**0.571**	**0.878**	0.681	0.786	0.721	**0.800**		0.664	0.251	**0.813**	0.652	0.780	0.161	0.752
	BERT^b^	0.780	0.560	0.757	**0.802**	**0.842**	**0.794**	0.775		**0.797**	**0.339**	0.750	**0.801**	**0.825**	**0.229**	**0.770**
	UniRep+BERT^b^	**0.782**	0.570	0.856	0.708	0.794	0.731	0.797		0.690	0.255	0.781	0.683	0.786	0.167	0.744

^a^The used ML algorithms were: LGBM: light gradient boosting machine; SVM: support vector machine; RF: random forest; LR: logistic regression; KNN: *k*-nearest neighbors; LDA: latent dirichlet allocation; NB: naive bayes.

^b^Feature vectors were created using UniRep: unified representation with 1900D; BERT: bidirectional encoder representation from transformers with 768D; UniRep+BERT: UniRep and BERT feature fusion vector with 2668D.

^c^Best performance values using the same ML model are shown in bold.

^d^Best performance values are shown in bold and are underlined.

In short, although there was a small improvement in the test result in some of the metrics when the fused vector set was used as input, the overall effect was not significant. For example, the fused feature model analyzed by LR outperformed the other vector sets on all 5-fold cross-validation and some of the independent test metrics (Sn: 4.55% - 15.01%, auROC: 0.20% - 6.36%, auPRC: 0.73% - 36.72%, F1: 2.38% - 10.49%). However, as apparent from Table 1, the high-dimensional fused features are likely to represent redundant information and further processing with most ML algorithms did not improve prediction performance but rather degraded it.

### Feature selection can improve predictive performance

**Figure 3:**
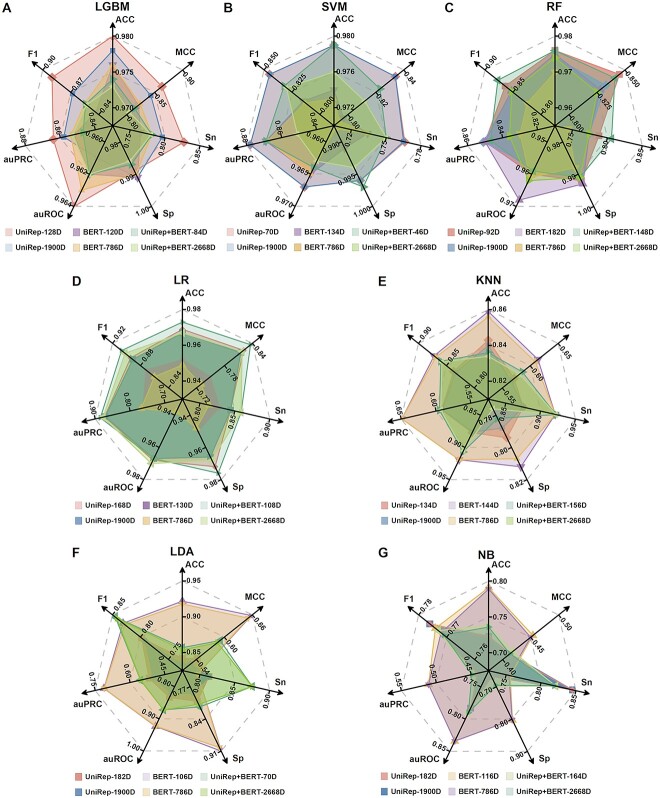
Comparison of model performance using the full or feature selected feature vectors. The results are obtained based on seven machine learning models, (**A**) LGBM, (**B**) SVM, (**C**) RF, (**D**) LR, (**E**) KNN, (**F**) LDA, and (**G**) NB. Numbers indicate the average of model performance in 5-fold cross-validation and independent test results.

Next, we sorted the features according to their importance using the LGBM algorithm. This step eliminated redundant and irrelevant features, while retaining significant ones. The results of 5-fold cross-validation and independent testing of models using the selected features are shown in Table 2. We also compared the developed models as shown in Figure 3, where the metrics shown represent the average of independent tests and the 5-fold cross-validation results.

**Figure 4:**
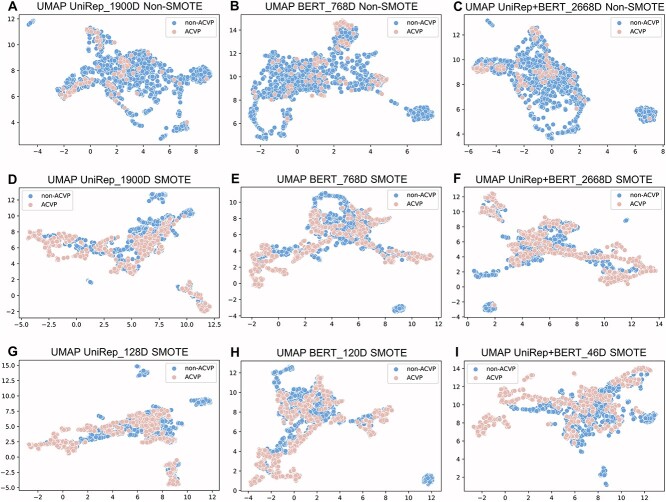
Visualization of the nine representative feature vector sets using the UMAP dimensionality reduction for feature representations. (**A**, **D**, **G**) are for the UniRep features, (**B**, **E**, **H**) are for the BERT feature, and (**C**, **F**, **I**) are for the UniRep+BERT feature.

**Table 2:** The results of 5-fold cross-validation and independent testing of feature selected feature vector extracted by UniRep and BERT

Model	Feature	Dim	5-fold cross-validation								Independent test						
			ACC	MCC	Sn	Sp	auROC	auPRC	F1		ACC	MCC	Sn	Sp	auROC	auPRC	F1
LGBM^a^	UniRep^b^	128	0.992	0.984	0.998	0.986	**1.000** ^c^	**1.000**	0.992		**0.967**	**0.742**	0.656	0.992	0.927	0.729	0.789
	BERT^b^	120	0.995	0.990	0.999	0.991	**1.000**	**1.000**	0.995		0.958	0.653	0.531	0.992	0.923	0.705	0.690
	UniRep+BERT^b^	84	0.993	0.986	**1.000**	0.986	**1.000**	**1.000**	0.993		0.956	0.641	0.563	0.987	0.924	0.694	0.714
SVM^a^	UniRep^b^	70	0.998	0.996	**1.000**	0.996	**1.000**	**1.000**	0.998		0.960	0.672	0.531	**0.995**	0.934	0.756	0.692
	BERT^b^	134	0.995	0.989	**1.000**	0.989	**1.000**	**1.000**	0.995		0.953	0.604	0.469	0.992	0.929	0.737	0.635
	UniRep+BERT^b^	46	**0.999**	**0.998**	**1.000**	**0.998**	**1.000**	**1.000**	**0.999**		0.958	0.649	0.500	**0.995**	0.927	0.738	0.664
RF^a^	UniRep^b^	92	0.986	0.972	0.982	0.990	0.999	0.999	0.986		0.965	0.717	0.594	**0.995**	0.920	0.701	0.743
	BERT^b^	182	0.990	0.979	0.986	0.993	0.999	1.000	0.990		0.960	0.676	0.563	0.992	0.937	0.700	0.717
	UniRep+BERT^b^	148	0.989	0.979	0.987	0.991	0.999	0.999	0.989		0.963	0.703	0.625	0.990	0.919	0.696	0.764
LR^a^	UniRep^b^	168	0.983	0.967	**1.000**	0.966	0.997	0.995	0.983		0.956	0.675	0.688	0.977	**0.939**	0.750	0.804
	BERT^b^	130	0.974	0.950	**1.000**	0.948	0.990	0.983	0.975		0.930	0.550	0.656	0.952	0.884	0.543	0.770
	UniRep+BERT^b^	108	0.987	0.975	**1.000**	0.975	0.999	0.999	0.988		0.958	0.696	0.719	0.977	0.935	**0.771**	0.825
KNN^a^	UniRep^b^	134	0.900	0.816	**1.000**	0.800	0.947	0.905	0.909		0.786	0.326	0.750	0.788	0.807	0.279	0.765
	BERT^b^	144	0.908	0.830	0.999	0.818	0.952	0.913	0.916		0.809	0.398	**0.844**	0.806	0.885	0.385	**0.828**
	UniRep+BERT^b^	156	0.896	0.810	**1.000**	0.793	0.940	0.893	0.906		0.776	0.362	**0.844**	0.771	0.873	0.297	0.814
LDA^a^	UniRep^b^	182	0.906	0.827	**1.000**	0.812	0.924	0.869	0.914		0.779	0.256	0.625	0.791	0.742	0.166	0.682
	BERT^b^	106	0.953	0.910	**1.000**	0.905	0.988	0.972	0.955		0.890	0.406	0.594	0.914	0.858	0.452	0.707
	UniRep+BERT^b^	70	0.910	0.834	**1.000**	0.819	0.937	0.892	0.917		0.804	0.347	0.750	0.809	0.807	0.219	0.773
NB^a^	UniRep^b^	182	0.781	0.574	0.878	0.684	0.786	0.721	0.801		0.664	0.251	0.813	0.652	0.780	0.161	0.752
	BERT^b^	116	0.784	0.569	0.762	0.805	0.842	0.793	0.779		0.797	0.339	0.750	0.801	0.825	0.229	0.770
	UniRep+BERT^b^	164	0.787	0.580	0.860	0.713	0.794	0.731	0.801		0.690	0.255	0.781	0.683	0.786	0.167	0.744

^a^The ML algorithms were: LGBM: light gradient boosting machine; SVM: support vector machine; RF: random forest; LR: logistic regression; KNN: *k*-nearest neighbors; LDA: latent dirichlet allocation; NB: naive bayes.

^b^Features were extracted using UniRep: unified representation; BERT: bidirectional encoder representation from transformers; UniRep+BERT: Fused UniRep and BERT feature vectors. The number of features (Dim column) was selected using LGBM.

^c^Best performance values are shown in bold and are underlined.

As these results indicate, irrespective of the ML method used, model performance improved with the use of selected features. As an example, when using LGBM (Figure 3A), the UniRep-128D model outperformed the UniRep-1900D model in 85.7% of the metrics (ACC: 0.14%, MCC: 2.35%, Sn: 3.88%, auROC: 0.27%, auPRC: 0.75%, F1: 2.61%). Thus, feature selection was an effective way of identifying important features and enhancing the performance of the ACVP prediction model.

### Visualization of feature representations

UMAP models the manifold with a fuzzy topology and finds embeddings by searching for low-dimensional projections of the data with the closest equivalent topology. The global structure of the data is maintained as much as possible. The visualization of the main feature models developed in this paper is shown in Figure 4.

Since, as mentioned before, our dataset was imbalanced (Figure 4B, C), such feature models tend to be misleading. Therefore, we performed a SMOTE-based data augmentation on the feature models (Figure 4D–F) so that non-ACVPs and ACVPs were not entirely isolated during visualization. As can be seen in Figure 4H and I, using selected features could separate non-ACVP and ACVP sequences better than the high-dimensional features without applied selection (Figure 4D–F).

### Comparing FEOpti-ACVP with previously reported selection strategies

In its final implementation, based on accuracy in independent test results, FEOpti-ACVP uses the top 128-dimensional features of UniRep data and combines this with ML analysis using the LGBM algorithm. The average results of 5-fold cross-validation and independent test for all SMOTE-balanced models are shown in Supplementary Table 2. As a final test, we compared the performance of this optimized version of FEOpti-ACVP with existing ACVP predictors, including PACVP [25], iACVP [20] and ENNAVIA-D [18]. The results of these comparisons using the dedicated test peptide dataset are shown in Table 3, while data on the 5-fold cross-validation are illustrated in Supplementary Table 3.

**Table 3:** Performance metrics FEOpti-ACVP and three previously reported ACVP predictor models, all tested on the same ACVP-IND dataset

Classifier	Independent test						
	ACC	MCC	Sn	Sp	auROC	auPRC	F1
FEOpti-ACVP	**0.967**	**0.742**	0.656	**0.992**	0.927	**0.729**	**0.789**
PACVP	0.921	0.614	0.875	0.924	**0.947**	/	0.622
iACVP	0.399	0.189	0.906	0.358	0.740	0.186	0.184
ENNAVIA-D	0.646	0.237	**1.000**	0.624	0.940	0.558	0.247

^a^Values indicating the best achieved performance are shown in bold and are underlined.

These results showed that FEOpti-ACVP could identify ACVP more effectively than previously reported approaches. The 5-fold cross-validation result showed that FEOpti-ACVP achieved ACC of 0.992, MCC of 0.984, Sn of 0.998, Sp of 0.986, auROC of 1.000, auPRC of 1.000, and the F1 was 0.992. The same values using the independent test dataset were: ACC 0.967 (an increase of 5.06–142.70% over competitors), MCC 0.742 (an increase of 20.72–292.54%), Sn 0.656, Sp 0.992 (an improvement by 7.36–177.45%), auROC 0.927, auPRC 0.729 (an increase by 30.65–292.27%), while F1 was 0.789 (a gain of 26.78–329.76%). These results demonstrated that FEOpti-ACVP could predict ACVPs more accurately and robustly than alternative solutions proposed to accomplish this task.

## CONCLUSION

This report describes the development of FEOpti-ACVP, a prediction model capable of identifying potential novel ACVPs. The results showed that feature extraction based on deep characterization learning was an effective strategy. The use of selected features, detected via the LGBM algorithm, represented an essential step in model building. The improved performance of the developed model following SMOTE-based data balancing was also evident, as shown by the dimensionality reduction visualization. In the final implementation, we chose to use the top 128-dimensional features from UniRep and combined these with the LGBM based ML algorithm to construct the final prediction model. Finally, when compared with existing ACVP prediction tools, FEOpti-ACVP had a superior ability to predict and identify effective ACVPs, achieving an ACC of 0.967 and a Sp of 0.992 on the independent test dataset. To make this tool available to the scientific community, we constructed a web server for FEOpti-ACVP (http://servers.aibiochem.net/soft/FEOpti-ACVP/). Here, the user only needs to enter the peptide sequence and click the run button to predict the probability of a peptide being a potential ACVP drug candidate. In addition, we hope that this prediction framework will have broader applications in bioinformatics.

**Key Points**

- The pretrained deep representation learning model for protein sequences is used for peptide sequence feature extraction.
- Compared with previous models combined with multiple artificial design features, FEOpti-ACVP used two types of features automatically extracted from peptide sequences.
- The independent test results of FEOpti-ACVP improved by 5.1-142.7% of accuracy to that of the reported best methods.
- A user-friendly webserver of FEOpti-ACVP is provided for readers.

## Supplementary Material

Supplementary_Material_1227_bbae037

## Data Availability

The benchmark dataset is available at http://public.aibiochem.net/peptides/FEOpti-ACVP/.

## Author Biographies

**Jici Jiang** is currently an undergraduate at the College of Biomedical Engineering, Sichuan University. Her research focuses on natural language processing and machine learning for bioinformatics.

**Hongdi Pei** is currently an undergraduate at the College of Biomedical Engineering, Sichuan University. His research interests include bioinformatics, medical imaging, and deep learning.

**Jiayu Li** is currently an undergraduate at the College of Life Science, Sichuan University. Her research interest is in computational biology.

**Mingxin Li** is currently a graduate student at the College of Biomedical Engineering, Sichuan University. Her research focuses on bioinformatics based on artificial intelligence.

**Quan Zou** is a professor in Institute of Fundamental and Frontier Sciences at University of Electronic Science and Technology of China. He received his PhD degree from Harbin Institute of Technology in 2009. He focuses on area of bio-sequences analysis.

**Zhibin Lv** is an associate professor at the College of Biomedical Engineering, Sichuan University. He received his PhD degree from Peking University in 2013. His research interest is on machine learning for bioinformatics.

## References

[1] Malone B , Urakova N, Snijder EJ, et al. Structures and functions of coronavirus replication–transcription complexes and their relevance for SARS-CoV-2 drug design. Nat Rev Mol Cell Biol 2022;23(1):21–39. 34824452 10.1038/s41580-021-00432-z PMC8613731

[2] Jin Z , Du X, Xu Y, et al. Structure of Mpro from SARS-CoV-2 and discovery of its inhibitors. Nature 2020;582(7811):289–93. 32272481 10.1038/s41586-020-2223-y

[3] Zhang Z , Cui F, Cao C, et al. Single-cell RNA analysis reveals the potential risk of organ-specific cell types vulnerable to SARS-CoV-2 infections. Comput Biol Med 2021;140:105092. 34864302 10.1016/j.compbiomed.2021.105092 PMC8628631

[4] Yang S , Wang Y, Chen Y, et al. MASQC: next generation sequencing assists third generation sequencing for quality control in N6-Methyladenine DNA identification. Front Genet 2020;11:269. 32269589 10.3389/fgene.2020.00269 PMC7109398

[5] Yang Z , Yi W, Tao J, et al. HPVMD-C: a disease-based mutation database of human papillomavirus in China. Database (Oxford) 2022;2022:baac018. 35348640 10.1093/database/baac018 PMC9216535

[6] Wang H , Guo F, Du M, et al. A novel method for drug-target interaction prediction based on graph transformers model. BMC Bioinformatics 2022;23(1):459. 36329406 10.1186/s12859-022-04812-w PMC9635108

[7] Thakur A , Sharma A, Alajangi HK, et al. In pursuit of next-generation therapeutics: antimicrobial peptides against superbugs, their sources, mechanism of action, nanotechnology-based delivery, and clinical applications. Int J Biol Macromol 2022;218:135–56. 35868409 10.1016/j.ijbiomac.2022.07.103

[8] Kim DI , Han SH, Park H, et al. Pseudo-isolated α-helix platform for the recognition of deep and narrow targets. J Am Chem Soc 2022;144(34):15519–28. 35972994 10.1021/jacs.2c03858

[9] Sharma A , Kaur M, Yadav P, et al. Expediting the drug discovery for ideal leads against SARS-CoV-2 via molecular docking of repurposed drugs. J Biomol Struct Dyn 2023;41(16):7949–65. 36165445 10.1080/07391102.2022.2127903

[10] Xia S , Wang L, Jiao F, et al. SARS-CoV-2 Omicron subvariants exhibit distinct fusogenicity, but similar sensitivity, to pan-CoV fusion inhibitors. Emerg Microbes Infect 2023;12(1):2178241. 36748716 10.1080/22221751.2023.2178241 PMC9970205

[11] Xue S , Wang X, Wang L, et al. A novel cyclic γ-AApeptide-based long-acting pan-coronavirus fusion inhibitor with potential oral bioavailability by targeting two sites in spike protein. Cell Discov 2022;8(1):88. 36075899 10.1038/s41421-022-00455-6 PMC9453727

[12] Xia S , Chan JF, Wang L, et al. Peptide-based pan-CoV fusion inhibitors maintain high potency against SARS-CoV-2 Omicron variant. Cell Res 2022;32(4):404–6. 35087243 10.1038/s41422-022-00617-x PMC8793821

[13] Lan Q , Chan JF, Xu W, et al. A palmitic acid-conjugated, peptide-based pan-CoV fusion inhibitor potently inhibits infection of SARS-CoV-2 Omicron and other variants of concern. Viruses 2022;14(3):549. 35336956 10.3390/v14030549 PMC8955410

[14] Duan Q , Xia S, Jiao F, et al. A modified fibronectin type III domain-conjugated, long-acting pan-coronavirus fusion inhibitor with extended half-life. Viruses 2022;14(4):655. 35458385 10.3390/v14040655 PMC9028128

[15] Yu L , Zheng YJ, Ju BY, et al. Research progress of miRNA-disease association prediction and comparison of related algorithms. Brief Bioinform 2022;23(3):bbac066. 35246678 10.1093/bib/bbac066

[16] Kaur M , Sharma A, Kumar S, et al. SARS-CoV-2: insights into its structural intricacies and functional aspects for drug and vaccine development. Int J Biol Macromol 2021;179:45–60. 33662418 10.1016/j.ijbiomac.2021.02.212 PMC7919520

[17] Pang Y , Wang Z, Jhong JH, et al. Identifying anti-coronavirus peptides by incorporating different negative datasets and imbalanced learning strategies. Brief Bioinform 2021;22(2):1085–95. 33497434 10.1093/bib/bbaa423 PMC7929366

[18] Timmons P , Hewage C. ENNAVIA is a novel method which employs neural networks for antiviral and anti-coronavirus activity prediction for therapeutic peptides. Brief Bioinform 2021;22(6):1–17. 34297817 10.1093/bib/bbab258 PMC8575049

[19] Tao J , Liu X, Yang S, et al. An efficient genomic signature ranking method for genomic island prediction from a single genome. J Theor Biol 2019;467:142–9. 30768974 10.1016/j.jtbi.2019.02.008

[20] Kurata H , Tsukiyama S, Manavalan B. iACVP: markedly enhanced identification of anti-coronavirus peptides using a dataset-specific word2vec model. Brief Bioinform 2022;23(4):1–15. 10.1093/bib/bbac265 35772910

[21] Dai Q , Bao C, Hai Y, et al. MTGIpick allows robust identification of genomic islands from a single genome. Brief Bioinform 2018;19(3):361–73. 28025178 10.1093/bib/bbw118 PMC6454522

[22] Kong R , Xu X, Liu X, et al. 2SigFinder: the combined use of small-scale and large-scale statistical testing for genomic island detection from a single genome. BMC Bioinformatics 2020;21(1):159. 32349677 10.1186/s12859-020-3501-2 PMC7191778

[23] Onesime M , Yang Z, Dai Q. Genomic island prediction via chi-square test and random forest algorithm. Comput Math Methods Med 2021;2021:9969751. 34122622 10.1155/2021/9969751 PMC8169257

[24] Dai Q , Ma S, Hai Y, et al. A segmentation based model for subcellular location prediction of apoptosis protein. Chemom Intel Lab Syst 2016;158:146–54.

[25] Chen S , Liao Y, Zhao J, et al. PACVP: prediction of anti-coronavirus peptides using a stacking learning strategy with effective feature representation. IEEE/ACM Trans Comput Biol Bioinform 2023;20(5):3106–16. 10.1109/TCBB.2023.3238370 37022025

[26] Liu M , Yang F, Xu Y. Identification of potential drug therapy for dermatofibrosarcoma protuberans with bioinformatics and deep learning technology. Curr Comput Aided Drug Des 2022;18(5):393–405. 35975851 10.2174/1573409918666220816112206

[27] Zhang S , Fan R, Liu Y, et al. Applications of transformer-based language models in bioinformatics: a survey. Bioinform Adv 2023;3(1):vbad001. 36845200 10.1093/bioadv/vbad001 PMC9950855

[28] Wang C , Zou Q. A machine learning method for differentiating and predicting human-infective coronavirus based on physicochemical features and composition of the spike protein. Chinese J Electron 2021;30(5):815–23.

[29] Wang Y , Zhai Y, Ding Y, et al. SBSM-pro: support bio-sequence machine for proteins. 2023. arXiv preprint arXiv:2308.10275.

[30] Yan K , Guo Y, Liu B. PreTP-2L: identification of therapeutic peptides and their types using two-layer ensemble learning framework. Bioinformatics 2023;39(4):btad125. 37010503 10.1093/bioinformatics/btad125 PMC10076046

[31] Kaur A , Chauhan APS, Aggarwal AK. Prediction of enhancers in DNA sequence data using a hybrid CNN-DLSTM model. IEEE/ACM Trans Comput Biol Bioinform 2023;20(2):1327–36. 35417351 10.1109/TCBB.2022.3167090

[32] He W , Jiang Y, Jin J, et al. Accelerating bioactive peptide discovery via mutual information-based meta-learning. Brief Bioinform 2022;23(1):bbab499. 34882225 10.1093/bib/bbab499

[33] Cao C , Kossinna P, Kwok D, et al. Disentangling genetic feature selection and aggregation in transcriptome-wide association studies. Genetics 2022;220(2):iyab216. 34849857 10.1093/genetics/iyab216 PMC9208638

[34] Cao C , Wang J, Kwok D, et al. webTWAS: a resource for disease candidate susceptibility genes identified by transcriptome-wide association study. Nucleic Acids Res 2022;50(D1):D1123–30. 34669946 10.1093/nar/gkab957 PMC8728162

[35] Zhang Z , Cui F, Lin C, et al. Critical downstream analysis steps for single-cell RNA sequencing data. Brief Bioinform 2021;22(5):bbab105. 33822873 10.1093/bib/bbab105

[36] Zhang Z , Cui F, Su W, et al. webSCST: an interactive web application for single-cell RNA-sequencing data and spatial transcriptomic data integration. Bioinformatics 2022;38(13):3488–9. 35604082 10.1093/bioinformatics/btac350

[37] Zhang Z , Cui F, Wang C, et al. Goals and approaches for each processing step for single-cell RNA sequencing data. Brief Bioinform 2021;22(4):bbaa314. 33316046 10.1093/bib/bbaa314

[38] Jiang J , Li J, Li J, et al. A machine learning method to identify umami peptide sequences by using multiplicative LSTM embedded features. Foods 2023;12(7):1498. 37048319 10.3390/foods12071498 PMC10094688

[39] Villegas-Morcillo A , Gomez AM, Sanchez V. An analysis of protein language model embeddings for fold prediction. Brief Bioinform 2022;23(3):bbac142. 35443054 10.1093/bib/bbac142

[40] Nourani E , Asgari E, McHardy AC, et al. TripletProt: deep representation learning of proteins based on Siamese networks. IEEE/ACM Trans Comput Biol Bioinform 2022;19(6):3744–53. 34460382 10.1109/TCBB.2021.3108718

[41] Jiang J , Lin X, Jiang Y, et al. Identify bitter peptides by using deep representation learning features. Int J Mol Sci 2022;23(14):7877. 35887225 10.3390/ijms23147877 PMC9315524

[42] Jiang L , Jiang J, Wang X, et al. IUP-BERT: identification of umami peptides based on BERT features. Foods 2022;11(22):3742. 36429332 10.3390/foods11223742 PMC9689418

[43] Zhang Q , Chen X, Li B, et al. A database of anti-coronavirus peptides. Sci Data 2022;9(1):294. 35697698 10.1038/s41597-022-01394-3 PMC9192597

[44] Chamoli T , Khera A, Sharma A, et al. Peptide Utility (PU) search server: a new tool for peptide sequence search from multiple databases. Heliyon 2022;8(12):e12283. 36590540 10.1016/j.heliyon.2022.e12283 PMC9800339

[45] Chawla NV , Bowyer KW, Hall LO, et al. SMOTE: synthetic minority over-sampling technique. J Artif Intell Res 2002;16:321–57.

[46] Alley EC , Khimulya G, Biswas S, et al. Unified rational protein engineering with sequence-based deep representation learning. Nat Methods 2019;16(12):1315–22. 31636460 10.1038/s41592-019-0598-1 PMC7067682

[47] Devlin J , Chang M-W, Lee K, et al. BERT: pre-training of deep bidirectional transformers for language understanding. In: Proceedings of the 2019 Conference of the North American Chapter of the Association for Computational Linguistics: Human Language Technologies, Minneapolis, Minnesota. Volume 1 (Long and Short Papers). Association for Computational Linguistics, 2019, p. 4171–86.

[48] Ahmed CM , Grams TR, Bloom DC, et al. Individual and synergistic anti-coronavirus activities of SOCS1/3 antagonist and interferon α1 peptides. Front Immunol 2022;13:902956. 35799776 10.3389/fimmu.2022.902956 PMC9254576

[49] Ao C , Ye X, Sakurai T, et al. m5U-SVM: identification of RNA 5-methyluridine modification sites based on multi-view features of physicochemical features and distributed representation. BMC Biol 2023;21(1):93. 37095510 10.1186/s12915-023-01596-0 PMC10127088

[50] Liang X , Li F, Chen J, et al. Large-scale comparative review and assessment of computational methods for anti-cancer peptide identification. Brief Bioinform 2021;22(4):bbaa312. 10.1093/bib/bbaa312 PMC8294543 33316035

[51] Chen L , Yu L, Gao L. Potent antibiotic design via guided search from antibacterial activity evaluations. Bioinformatics 2023;39(2):btad059. 36707990 10.1093/bioinformatics/btad059 PMC9897189

[52] Yu L , Xia MF, An Q. A network embedding framework based on integrating multiplex network for drug combination prediction. Brief Bioinform 2022;23(1):bbab364. 34505623 10.1093/bib/bbab364

[53] Jin J , Yu Y, Wei L. Mouse4mC-BGRU: deep learning for predicting DNA N4-methylcytosine sites in mouse genome. Methods 2022;204:258–62. 35093537 10.1016/j.ymeth.2022.01.009

[54] Wang R , Jiang Y, Jin J, et al. DeepBIO: an automated and interpretable deep-learning platform for high-throughput biological sequence prediction, functional annotation, and visualization analysis. Nucleic Acids Res 2023;51(7):3017–29. 36796796 10.1093/nar/gkad055 PMC10123094

[55] Jin J , Yu Y, Wang R, et al. iDNA-ABF: multi-scale deep biological language learning model for the interpretable prediction of DNA methylations. Genome Biol 2022;23(1):1–23. 36253864 10.1186/s13059-022-02780-1 PMC9575223

