# Supplementary material for: FEOpti-ACVP: identification of novel anti-coronavirus peptide sequences based on feature engineering and optimization
Source: Brief Bioinform. 2024 Feb 14;25(2):bbae037. doi: 10.1093/bib/bbae037 (PMC10939380; doi:10.1093/bib/bbae037)
Supplement: Supplementary_Material_1227_bbae037 [file supplementary_material_1227_bbae037.docx]

**Supplementary table legends:**

**Supplementary Table 1.** Results of 5-fold cross-validation and independent testing of the seven machine learning (ML) models balanced with synthetic minority over-sampling technique (SMOTE) and without SMOTE.

| Feature | Model | SMOTE | 5-fold cross-validation | | | | | | Independent test | | | | | |
| --- | --- | --- | --- | --- | --- | --- | --- | --- | --- | --- | --- | --- | --- | --- |
|  |  |  | ACC | MCC | Sn | Sp | auROC | auPRC | ACC | MCC | Sn | Sp | auROC | auPRC |
| UniRep^b^  (1900D) | LGBM^c^ | NO | 0.948 | 0.520 | 0.296 | 0.999 | 0.875 | 0.593 | 0.951 | 0.574 | 0.406 | 0.995 | 0.928 | 0.733 |
|  |  | YES | 0.994 | 0.987 | 0.999 | 0.989 | **1.000^a^** | **1.000** | **0.963** | **0.698** | 0.594 | 0.992 | 0.922 | 0.716 |
|  | SVM^c^ | NO | 0.953 | 0.576 | 0.400 | 0.996 | 0.876 | 0.664 | **0.963** | 0.695 | 0.563 | 0.995 | **0.945** | **0.809** |
|  |  | YES | **0.998** | **0.996** | 0.999 | 0.996 | **1.000** | **1.000** | 0.960 | 0.672 | 0.531 | 0.995 | 0.934 | 0.756 |
|  | RF^c^ | NO | 0.945 | 0.479 | 0.248 | **1.000** | 0.850 | 0.527 | 0.946 | 0.516 | 0.281 | **1.000** | 0.921 | 0.681 |
|  |  | YES | 0.986 | 0.971 | 0.985 | 0.986 | 0.999 | 0.999 | **0.963** | 0.695 | 0.563 | 0.995 | 0.918 | 0.704 |
|  | KNN^c^ | NO | 0.942 | 0.501 | 0.416 | 0.983 | 0.829 | 0.485 | 0.949 | 0.578 | 0.500 | 0.985 | 0.820 | 0.563 |
|  |  | YES | 0.894 | 0.806 | **1.000** | 0.787 | 0.946 | 0.902 | 0.786 | 0.326 | 0.750 | 0.788 | 0.807 | 0.279 |
|  | LDA^c^ | NO | 0.791 | 0.192 | 0.480 | 0.815 | 0.639 | 0.142 | 0.783 | 0.261 | 0.625 | 0.796 | 0.750 | 0.174 |
|  |  | YES | 0.888 | 0.797 | **1.000** | 0.777 | 0.901 | 0.835 | 0.779 | 0.256 | 0.625 | 0.791 | 0.742 | 0.166 |
|  | NB^c^ | NO | 0.668 | 0.265 | 0.840 | 0.655 | 0.752 | 0.156 | 0.655 | 0.304 | **0.938** | 0.632 | 0.792 | 0.171 |
|  |  | YES | 0.780 | 0.571 | 0.878 | 0.681 | 0.786 | 0.721 | 0.664 | 0.251 | 0.813 | 0.652 | 0.780 | 0.161 |
|  | LR^c^ | NO | 0.946 | 0.502 | 0.304 | 0.997 | 0.863 | 0.575 | 0.946 | 0.515 | 0.313 | 0.998 | 0.944 | 0.734 |
|  |  | YES | 0.978 | 0.956 | **1.000** | 0.955 | 0.995 | 0.993 | 0.956 | 0.675 | 0.688 | 0.977 | 0.940 | 0.750 |
| BERT^b^  (768D) | LGBM^c^ | NO | 0.948 | 0.516 | 0.296 | **0.999** | 0.855 | 0.561 | 0.949 | 0.547 | 0.375 | 0.995 | 0.937 | 0.702 |
|  |  | YES | **0.995** | **0.989** | 0.999 | 0.991 | **1.000** | **1.000** | **0.958** | **0.658** | 0.563 | 0.990 | 0.926 | 0.708 |
|  | SVM^c^ | NO | 0.953 | 0.586 | 0.416 | 0.995 | 0.870 | 0.636 | **0.958** | 0.647 | 0.469 | 0.998 | **0.938** | **0.784** |
|  |  | YES | 0.992 | 0.984 | **1.000** | 0.984 | **1.000** | **1.000** | 0.953 | 0.604 | 0.469 | 0.992 | 0.929 | 0.737 |
|  | RF^c^ | NO | 0.945 | 0.474 | 0.256 | **0.999** | 0.829 | 0.517 | 0.946 | 0.516 | 0.281 | **1.000** | 0.903 | 0.611 |
|  |  | YES | 0.988 | 0.977 | 0.989 | 0.988 | 0.999 | 0.999 | 0.951 | 0.594 | 0.500 | 0.987 | 0.923 | 0.661 |
|  | KNN^c^ | NO | 0.947 | 0.506 | 0.304 | 0.997 | 0.807 | 0.454 | 0.951 | 0.571 | 0.344 | **1.000** | 0.930 | 0.625 |
|  |  | YES | 0.902 | 0.820 | 0.997 | 0.807 | 0.952 | 0.913 | 0.809 | 0.398 | **0.844** | 0.806 | 0.885 | 0.385 |
|  | LDA^c^ | NO | 0.901 | 0.373 | 0.488 | 0.934 | 0.804 | 0.406 | 0.930 | 0.536 | 0.625 | 0.955 | 0.866 | 0.556 |
|  |  | YES | 0.945 | 0.896 | **1.000** | 0.890 | 0.987 | 0.971 | 0.890 | 0.406 | 0.594 | 0.914 | 0.858 | 0.452 |
|  | NB^c^ | NO | 0.778 | 0.232 | 0.584 | 0.793 | 0.767 | 0.187 | 0.795 | 0.336 | 0.750 | 0.799 | 0.839 | 0.227 |
|  |  | YES | 0.780 | 0.560 | 0.757 | 0.802 | 0.842 | 0.794 | 0.797 | 0.339 | 0.750 | 0.801 | 0.825 | 0.229 |
|  | LR^c^ | NO | 0.942 | 0.426 | 0.208 | **0.999** | 0.837 | 0.495 | 0.944 | 0.485 | 0.281 | 0.998 | 0.915 | 0.614 |
|  |  | YES | 0.974 | 0.950 | **1.000** | 0.948 | 0.990 | 0.983 | 0.923 | 0.511 | 0.625 | 0.947 | 0.885 | 0.553 |
| UniRep  +BERT^b^  (2668D) | LGBM^c^ | NO | 0.949 | 0.528 | 0.304 | 0.999 | 0.889 | 0.611 | 0.949 | 0.544 | 0.344 | 0.998 | 0.931 | 0.725 |
|  |  | YES | 0.992 | 0.985 | 0.998 | 0.987 | **1.000** | **1.000** | 0.953 | 0.626 | 0.563 | 0.985 | 0.925 | 0.706 |
|  | SVM^c^ | NO | 0.950 | 0.543 | 0.328 | 0.999 | 0.906 | 0.670 | 0.958 | 0.649 | 0.500 | 0.995 | 0.942 | **0.780** |
|  |  | YES | **0.997** | **0.994** | 0.999 | 0.995 | **1.000** | **1.000** | 0.956 | 0.629 | 0.500 | 0.992 | 0.926 | 0.703 |
|  | RF^c^ | NO | 0.945 | 0.482 | 0.248 | **1.000** | 0.845 | 0.525 | 0.944 | 0.486 | 0.250 | **1.000** | 0.912 | 0.644 |
|  |  | YES | 0.987 | 0.975 | 0.985 | 0.990 | 0.999 | 0.999 | **0.960** | **0.676** | 0.563 | 0.992 | 0.926 | 0.673 |
|  | KNN^c^ | NO | 0.943 | 0.461 | 0.264 | 0.997 | 0.869 | 0.501 | 0.949 | 0.544 | 0.344 | 0.998 | 0.938 | 0.628 |
|  |  | YES | 0.891 | 0.801 | **1.000** | 0.782 | 0.937 | 0.889 | 0.776 | 0.362 | 0.844 | 0.771 | 0.873 | 0.297 |
|  | LDA^c^ | NO | 0.814 | 0.255 | 0.536 | 0.835 | 0.709 | 0.205 | 0.821 | 0.367 | 0.750 | 0.826 | 0.812 | 0.225 |
|  |  | YES | 0.904 | 0.823 | **1.000** | 0.807 | 0.938 | 0.890 | 0.804 | 0.347 | 0.750 | 0.809 | 0.807 | 0.219 |
|  | NB^c^ | NO | 0.696 | 0.270 | 0.808 | 0.687 | 0.753 | 0.160 | 0.678 | 0.306 | **0.906** | 0.660 | 0.800 | 0.177 |
|  |  | YES | 0.782 | 0.570 | 0.856 | 0.708 | 0.794 | 0.731 | 0.690 | 0.255 | 0.781 | 0.683 | 0.786 | 0.167 |
|  | LR^c^ | NO | 0.947 | 0.508 | 0.288 | 0.999 | 0.861 | 0.549 | 0.946 | 0.515 | 0.313 | 0.998 | **0.944** | 0.716 |
|  |  | YES | 0.981 | 0.964 | **1.000** | 0.963 | 0.999 | 0.998 | 0.953 | 0.672 | 0.719 | 0.972 | 0.941 | 0.756 |

^a^Best performance values for the same feature are indicated in bold and underlined.

^b^UniRep: unified representation with 1900D; BERT: bidirectional encoder representation from transformers with 768D; UniRep+BERT: UniRep and BERT feature fusion with 2668D.

^c^LGBM: light gradient boosting machine; SVM: support vector machine; RF: random forest; LR: logistic regression; KNN: k-nearest neighbors; LDA: latent dirichlet allocation; NB: naive bayes.

**Supplementary Table 2.** The average of 5-fold cross-validation and independent testing of seven ML models with SMOTE

| Model | Feature | Dim | Average Value | | | | | | |
| --- | --- | --- | --- | --- | --- | --- | --- | --- | --- |
|  |  |  | ACC | MCC | Sn | Sp | auROC | auPRC | F1 |
| LGBM^c^ | UniRep^b^ | 1900 | 0.978 | 0.843 | 0.796 | 0.991 | 0.961 | 0.858 | 0.868 |
|  |  | 128 | **0.980^a^** | **0.863** | 0.827 | 0.989 | 0.964 | 0.864 | 0.890 |
|  | BERT^b^ | 768 | 0.976 | 0.824 | 0.781 | 0.990 | 0.963 | 0.854 | 0.855 |
|  |  | 120 | 0.976 | 0.821 | 0.765 | 0.991 | 0.962 | 0.852 | 0.843 |
|  | UniRep+BERT^b^ | 2668 | 0.973 | 0.805 | 0.780 | 0.986 | 0.962 | 0.853 | 0.853 |
|  |  | 84 | 0.974 | 0.814 | 0.781 | 0.987 | 0.962 | 0.847 | 0.854 |
| SVM^c^ | UniRep^b^ | 1900 | 0.979 | 0.834 | 0.765 | 0.996 | 0.967 | 0.878 | 0.845 |
|  |  | 70 | 0.979 | 0.834 | 0.766 | 0.996 | 0.967 | 0.878 | 0.845 |
|  | BERT^b^ | 768 | 0.973 | 0.794 | 0.734 | 0.988 | 0.965 | 0.868 | 0.813 |
|  |  | 134 | 0.974 | 0.797 | 0.734 | 0.991 | 0.965 | 0.869 | 0.815 |
|  | UniRep+BERT^b^ | 2668 | 0.976 | 0.812 | 0.750 | 0.994 | 0.963 | 0.851 | 0.830 |
|  |  | 46 | 0.979 | 0.823 | 0.750 | **0.997** | 0.964 | 0.869 | 0.832 |
| RF^c^ | UniRep^b^ | 1900 | 0.974 | 0.833 | 0.774 | 0.991 | 0.958 | 0.851 | 0.852 |
|  |  | 92 | 0.976 | 0.845 | 0.788 | 0.992 | 0.960 | 0.850 | 0.864 |
|  | BERT^b^ | 768 | 0.970 | 0.785 | 0.744 | 0.988 | 0.961 | 0.830 | 0.825 |
|  |  | 182 | 0.975 | 0.828 | 0.774 | 0.993 | 0.968 | 0.850 | 0.853 |
|  | UniRep+BERT^b^ | 2668 | 0.974 | 0.825 | 0.774 | 0.991 | 0.962 | 0.836 | 0.852 |
|  |  | 148 | 0.976 | 0.841 | 0.806 | 0.991 | 0.959 | 0.847 | 0.877 |
| KNN^c^ | UniRep^b^ | 1900 | 0.840 | 0.566 | 0.875 | 0.788 | 0.876 | 0.591 | 0.834 |
|  |  | 134 | 0.843 | 0.571 | 0.875 | 0.794 | 0.877 | 0.592 | 0.837 |
|  | BERT^b^ | 768 | 0.856 | 0.609 | 0.921 | 0.807 | 0.919 | 0.649 | 0.869 |
|  |  | 144 | 0.859 | 0.614 | 0.921 | 0.812 | 0.919 | 0.649 | 0.872 |
|  | UniRep+BERT^b^ | 2668 | 0.834 | 0.581 | **0.922** | 0.776 | 0.905 | 0.593 | 0.858 |
|  |  | 156 | 0.836 | 0.586 | **0.922** | 0.782 | 0.907 | 0.595 | 0.860 |
| LDA^c^ | UniRep^b^ | 1900 | 0.834 | 0.527 | 0.813 | 0.784 | 0.821 | 0.501 | 0.791 |
|  |  | 182 | 0.842 | 0.542 | 0.813 | 0.802 | 0.833 | 0.518 | 0.798 |
|  | BERT^b^ | 768 | 0.918 | 0.651 | 0.797 | 0.902 | 0.922 | 0.711 | 0.828 |
|  |  | 106 | 0.922 | 0.658 | 0.797 | 0.910 | 0.923 | 0.712 | 0.831 |
|  | UniRep+BERT^b^ | 2668 | 0.854 | 0.585 | 0.875 | 0.808 | 0.872 | 0.554 | 0.842 |
|  |  | 70 | 0.857 | 0.591 | 0.875 | 0.814 | 0.872 | 0.555 | 0.845 |
| NB^c^ | UniRep^b^ | 1900 | 0.722 | 0.411 | 0.845 | 0.667 | 0.783 | 0.441 | 0.776 |
|  |  | 182 | 0.723 | 0.413 | 0.845 | 0.668 | 0.783 | 0.441 | 0.776 |
|  | BERT^b^ | 768 | 0.789 | 0.450 | 0.754 | 0.802 | 0.834 | 0.511 | 0.772 |
|  |  | 116 | 0.791 | 0.454 | 0.756 | 0.803 | 0.834 | 0.511 | 0.774 |
|  | UniRep+BERT^b^ | 2668 | 0.736 | 0.413 | 0.819 | 0.695 | 0.790 | 0.449 | 0.771 |
|  |  | 164 | 0.738 | 0.418 | 0.821 | 0.698 | 0.790 | 0.449 | 0.773 |
| LR^c^ | UniRep^b^ | 1900 | 0.967 | 0.815 | 0.844 | 0.966 | 0.967 | 0.871 | 0.891 |
|  |  | 168 | 0.969 | 0.821 | 0.844 | 0.972 | 0.968 | 0.873 | 0.894 |
|  | BERT^b^ | 768 | 0.949 | 0.730 | 0.813 | 0.948 | 0.938 | 0.768 | 0.860 |
|  |  | 130 | 0.952 | 0.750 | 0.828 | 0.950 | 0.937 | 0.763 | 0.872 |
|  | UniRep+BERT^b^ | 2668 | 0.967 | 0.818 | 0.859 | 0.968 | **0.970** | 0.877 | 0.902 |
|  |  | 108 | 0.973 | 0.836 | 0.859 | 0.976 | 0.967 | **0.885** | **0.907** |

^a^Best performance values are indicated in bold and underlined.

^b^UniRep: unified representation; BERT: bidirectional encoder representation; UniRep+BERT: UniRep and BERT feature fusion.

^c^LGBM: light gradient boosting machine; SVM: support vector machine; RF: random forest; LR: logistic regression; KNN: k-nearest neighbors; LDA: latent dirichlet allocation; NB: naive bayes.

**Supplementary Table 3.** 5-fold cross-validation results for FEOpti-ACVP

| Classifier | 5-fold cross-validation | | | | | | |
| --- | --- | --- | --- | --- | --- | --- | --- |
|  | ACC | MCC | Sn | Sp | auROC | auPRC | F1 |
| FEOpti-ACVP | 0.992 | 0.984 | 0.998 | 0.986 | 1.000 | 1.000 | 0.992 |
